# Supplementary material for: Intense light as anticoagulant therapy in humans
Source: PLoS One. 2020 Dec 31;15(12):e0244792. doi: 10.1371/journal.pone.0244792 (PMC7775081; doi:10.1371/journal.pone.0244792)
Supplement: S1 Fig — Serum troponin-I from Per2loxP/loxP-VE Cadherin Cre (endothelial specific), Per2loxP/loxP-Myosin Cre (cardiomyocyte sepcfic), Per2loxP/loxP-Lyz2 Cre (bone marrow specific) after 60 min of in situ myocardial ischemia and 2h reperfusion (mean±SD; n = 5). (PDF) [file pone.0244792.s001.pdf]

S1 Fig

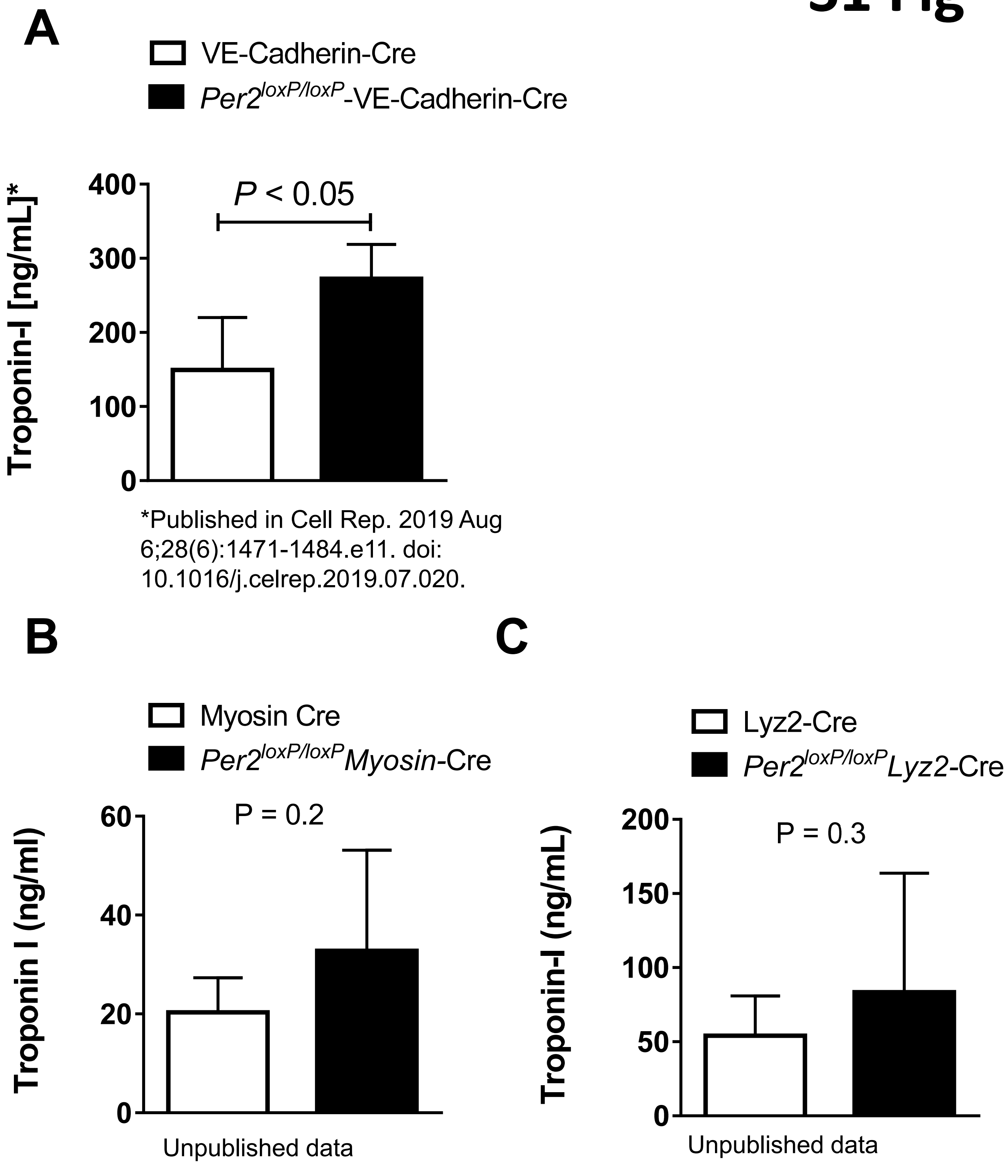

S1 Fig. Serum troponin-I from *Per2*$^{loxP/loxP}$-VE Cadherin Cre (endothelial specific), *Per2*$^{loxP/loxP}$-Myosin Cre (cardiomyocyte sepcfic), *Per2*$^{loxP/loxP}$-Lyz2 Cre (bone marrow specific) after 60 min of in situ myocardial ischemia and 2h reperfusion (mean±SD; n=5).
